# Dry Spells and Extreme Precipitation are The Main Trigger of Landslides in Central Europe

**DOI:** 10.1038/s41598-019-51148-2

**Published:** 2019-10-10

**Authors:** Radek Tichavský, Juan Antonio Ballesteros-Cánovas, Karel Šilhán, Radim Tolasz, Markus Stoffel

**Affiliations:** 1 Department of Physical Geography and Geoecology, Faculty of Science, University of Ostrava, Chittussiho 10, 71000 Ostrava, Czech Republic; 2 Climate Change Impacts and Risks in the Anthropocene (C-CIA), Institute for Environmental Sciences, University of Geneva, 66 Boulevard Carl-Vogt, 1205 Geneva, Switzerland; 3 Dendrolab.ch, Department of Earth Sciences, University of Geneva, 13 rue des Maraîchers, 1205 Geneva, Switzerland; 4 Czech Hydrometeorological Institute, Na Šabatce 17, 143 06 Praha 4 -, Komořany, Czech Republic; 5 Department F.-A. Forel for Environmental and Aquatic Sciences, University of Geneva, 66 Boulevard Carl-Vogt, 1205 Geneva, Switzerland

**Keywords:** Climate and Earth system modelling, Natural hazards, Geomorphology

## Abstract

Landslides are frequently triggered by extreme meteorological events which has led to concern and debate about their activity in a future greenhouse climate. It is also hypothesized that dry spells preceding triggering rainfall may increase slope predisposition to sliding, especially in the case of clay-rich soils. Here we combined dendrogeomorphic time series of landslides and climatic records to test the possible role of dry spells and extreme downpours on process activity in the Outer Western Carpathians (Central Europe). To this end, we tested time series of past frequencies and return periods of landslide reactivations at the regional scale with a Generalized Linear Mixed (GLM) model to explore linkages between landslide occurrences and triggering climate variables. Results show that landslide reactivations are concentrated during years in which spring and summer precipitation sums were significantly higher than usual, and that triggering mechanisms vary between different types of landslides (i.e. complex, shallow or flow-like). The GLM model also points to the susceptibility of landslide bodies to the combined occurrence of long, dry spells followed by large precipitation. Such situations are likely to increase in frequency in the future as climate models predict an enhancement of heatwaves and dry spells in future summers, that would be interrupted by less frequent, yet more intense storms, especially also in mountain regions.

## Introduction

Landslides represent global natural hazards causing thousands of fatalities every year and heavy devastation of infrastructure^1^. In the most affected areas, financial costs and countermeasures are in the order of billions of dollars^2,3^. Whereas lithology, slope morphology, land-use changes, climate warming, and/or anthropogenic pressure can predispose slope stability^4–8^, it is extreme rainfall, rapid snowmelt or earthquakes that will ultimately trigger landslide events^9^. In addition, the timing and velocity of slowly/persistently moving earthflows can be driven by internal conditions within the landslide body (such as soil swelling) and less so climatic triggers^10^. A proper understanding of the interactions and interdependencies of these factors and how climatic events influence the occurrence of landslides is crucial to understand landslide frequencies and magnitudes, even more so in a context of climate change where triggering conditions are likely to change.

Landslide research has recently focused on the monitoring of their movement using either *in-situ*^11^ or remotely sensed techniques^12^, calculation of rainfall intensity-duration thresholds for the initiation of mass movement activity^13^, landscape modelling and surface roughness dating^14^, and geochronological approaches to determine the age of landslide bodies or their activity. If combined with historical sources, geochronological data and palaeoclimatic proxies have repeatedly evidenced linkages between climate and landslide activity^15^. It was shown that both long-lasting^16–19^ and short-term, yet intense rainfalls^19–22^ are key drivers of landsliding, along with rapid snowmelt episodes or rain-on-snow events^23–25^. Besides, the combined effect of dry spells and extreme rainfall has been linked to landslide activity due to shrinking-swelling effects of clayey minerals in the weathered bedrock of landslide bodies^10,26^. In this context, the current understanding is that macropores of clay-rich soils would enlarge during dry spells which would then facilitate water transport into the landslide body during subsequent, high-magnitude rainfall^26^.

An improved understanding of how the consecutive occurrence of dry spells and downpours impacts on landslide dynamics are quite central because IPCC scenarios^27^ across Europe project an increase in the intensity and duration of dry spells/heatwaves and an increase in the intensity of heavy rainfall events. The combined occurrence of both phenomena – dry spell duration and precipitation intensity – is referred to as hydroclimatic intensity and shown to have increased in response to global warming over the second half of the 20^th^ century^28^.

The actual triggering of landslides also depends on the structural setting and lithology which in turn may differ among different landslide types: deep-seated landslides are generally thought to respond to excessive monthly and/or seasonal rainfall sums, whereas shallow landsliding would depend mainly on intense, yet short-lived rainfall peaks^5,29^.

To test the hypotheses of changes in hydroclimatic intensity and differences in triggers, robust, long-term records of landslide activity are critically needed. In reality, however, assessments are often hampered by fairly incomplete, descriptive records of historical activity on one hand, or short periods covered by systematic monitoring limiting the number of recorded landslides on the other hand, as well as by the absence of systematic, long-term meteorological data^6^. The extension of times series of landslides and the accurate dating of landslide activity is thus crucial for any analysis of climate factors triggering events. Besides the application of surface roughness dating^14^ and satellite imagery interpretation^29^, dendrogeomorphology (i.e. the dating of geomorphic processes using tree-ring records^30^) can overcome this shortcoming by dating past landslide activity in forested regions^16,23,31,32^. The resulting chronologies may be used as a basis for the comparison of climate records with years or decades with enhanced mass movement activity^33,34^. In addition, by modelling the combined effect of climate variables, one may also gain deeper understanding of mass movement triggering mechanisms^23,35^.

In the past, however, research on climate–landslide relations focused at the local or catchment scales^32,36–39^ whereas regional, tree-ring based chronologies of past landsliding have remained scarce. First promising attempts towards regional analysis were realized on seven landslide bodies in the Pyrenees and French Alps to reveal common mechanisms of landslide triggering^16,23^.

Here, we use a comprehensive regional, dendrogeomorphic landslide reconstruction^40^ from the Outer Western Carpathians (Central Europe) to test whether the successive occurrence of dry spells and extreme precipitation has had a negative effect on landslide activity in the past. To this end, we combine reconstructed time series from 26 landslide bodies with climate variables using a Generalized Linear Mixed (GLM) model, and will put observed, past climate-landslide drivers into perspective by comparing outcomes with projections of future climate change.

### Landslide region

**Figure 1:**
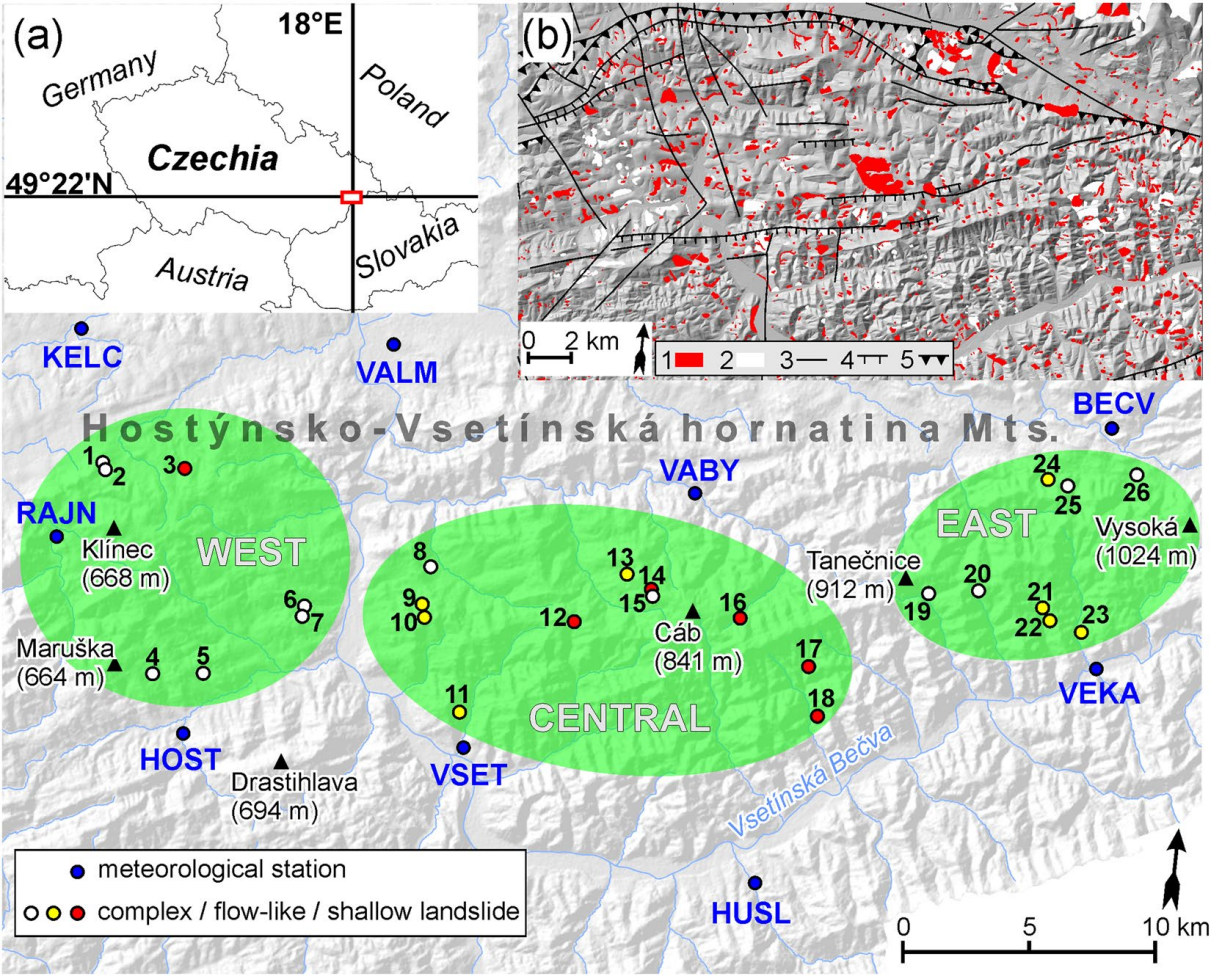
Location of landslide sites and meteorological stations. Map (**a**) shows the location of the study region within Central Europe. Map (**b**) shows the distribution of known landslides and tectonic features^75,76^: 1 – active landslides; 2 – non-active landslides; 3 – normal fault; 4 – thrust fault; 5 – fold nappe. Maps were created and modified in the ArcMap 10.1 software (ESRI; https://www.esri.com/en-us/home). Source of base maps: images of SRTM 1 Arc-Second Global^77^ available from USGS Earth Explorer (https://earthexplorer.usgs.gov).

The Hostýnsko-Vsetínská hornatina Mts. represent a mid-mountain part of the Outer Western Carpathians (Central Europe) and are centred around 49.4°N and 18.0°E (Fig. 1). The area is composed of Palaeogene flysch (predominantly sandstone, claystone, and shale) thrusted to nappe structures with evidence of multiple, past tectonic, but without tectonic seismic activity in modern times. The presence of montmorillonitic clays (e.g., smectite) favours the shrinking and swelling of weathered bedrock, thereby the cohesion and friction angle^41^. This lithological setting and mineralogical composition has favoured the evolution of different landslide types in the region (i.e. shallow, flow-like, and complex landslides) and the formation of very distinct morphological features (Supplementary Figs S1, S2). In fact, the density and variety of different, active landslides is considered unique for Central Europe^42^.

The area of interest (*c*. 600 km^2^; 40 × 15 km) is characterized by fully humid temperate climate. Mean annual temperature ranges from 5 °C at the summits to 8 °C in the valleys. Precipitation increases in zonal direction with the lowest annual precipitation in the western part (700–800 mm) and highest values in the eastern part (900–1200 mm) of the study region. Summers normally receive the largest amounts of rainfall (Fig. S3) when 24-h rainfall sums may exceed 100 mm in extreme cases. Snow covers the surface between December and April and lasts on the ground for 60 days at the foothills to 140 days at summits^43^.

The region suffered from intense deforestation for pasturing during the Wallachian colonisation in the 15^th^ to 17^th^ centuries. Nowadays, forest stands cover the slopes again, and are composed predominantly of introduced Norway spruce (*Picea abies* (L.) Karst.) with occasional silver fir (*Abies alba* Mill.), European larch (*Larix decidua* Mill.) and European beech (*Fagus sylvatica* L.) trees.

The presence of non-permeable, plastic claystone and extreme precipitation during springs and summers favours the release of frequent landslide activity over the entire region. Documentary sources^44–46^ report on landslide calamites in 1919, 1940, and 1997 as a result of extreme rainfall episodes.

## Results

### Precipitation triggers of the landslide activity

**Figure 2:**
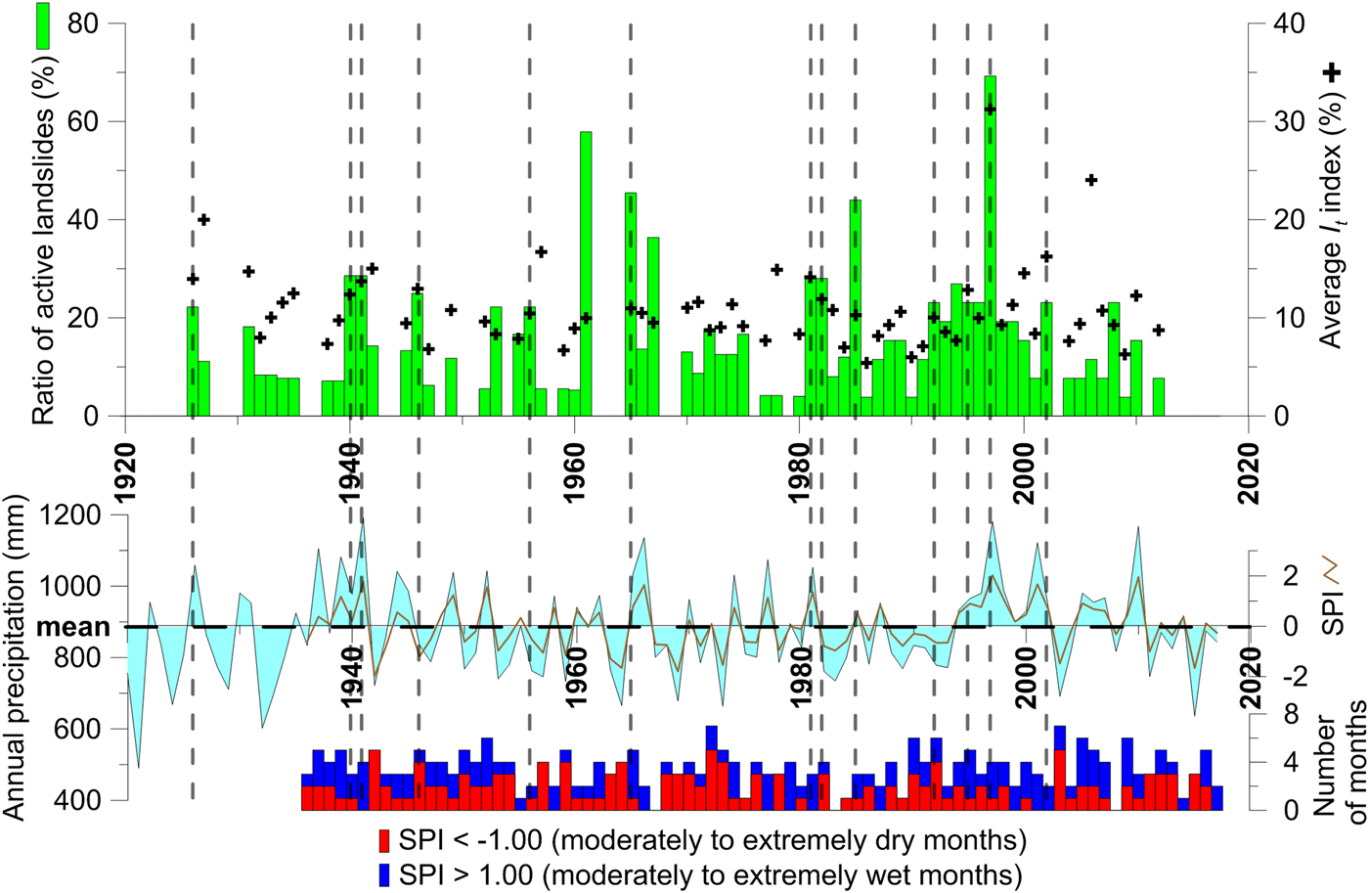
Chronology of landslide activity, annual precipitation totals in the study region, annual Standardized Precipitation Index (SPI) and number of wet/dry months according to the monthly SPI. The ratio of active landslides and value of average *I*_*t*_ index (i.e. number of disturbed trees in a particular year divided by the number of all sampled trees living in that particular year; in %) of all landslide events^40^ is denoted for each event year. Grey dashed lines show the event years with more than 20% of active landslides and exceeding the 10% value of *I*_*t*_ index.

**Figure 3:**
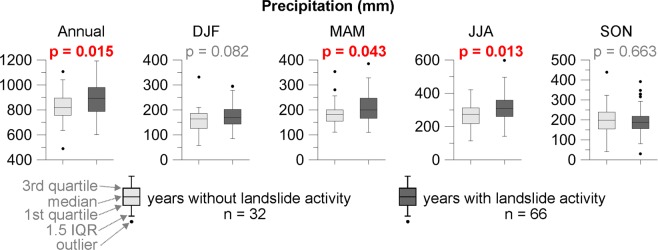
Comparison of years with and without the landslide activity based on annual and seasonal precipitation. IQR = Interquartile range.

Dendrogeomorphic reconstruction of past process activity provides evidence of 237 landslide events over the 26 landslide bodies for the period 1920–2017 (Fig. 2). Landslides occurred in 66 different years of which 51.5% were characterized by above-average annual precipitation. Years with landslide reactivations show significantly higher annual precipitation sums than those without landslide activity (p = 0.02), and even more so when summer (p = 0.01) or spring (p = 0.04) precipitation sums are taken into consideration. By contrast, autumn and winter precipitation do not significantly differ between event and non-event years (p = 0.66 and 0.08, respectively; Fig. 3). The annual values of the Standardized Precipitation Index (SPI; calculated for the period 1936–2017; Fig. 2) point to eleven landslide reactivations that would have occurred during moderately or extremely wet years (e.g. 1941, 1981, 1997), whereas seven landslide events occurred during moderately or extremely dry years (e.g. 1942, 1959, 1973). In addition, monthly SPI allowed identification of wetting and drying cycles during particular years (Fig. S4). We observed the occurrence of drying cycles in winter and/or spring followed by extreme rainfall event in summer and/or autumn in 50% of the years (e.g. 1939–40, 1956–57, 1970–72, 1982, 1994, 1996–97) in 50% of the landslides for which the *I*_*t*_ index >10% and/or the ratio of landslides exceeded 10%. The remaining events were either associated to rainfall accumulation over the course of the year, possible rain-on-snow events, or could not be associated to any specific pattern (Fig. S4).

**Figure 4:**
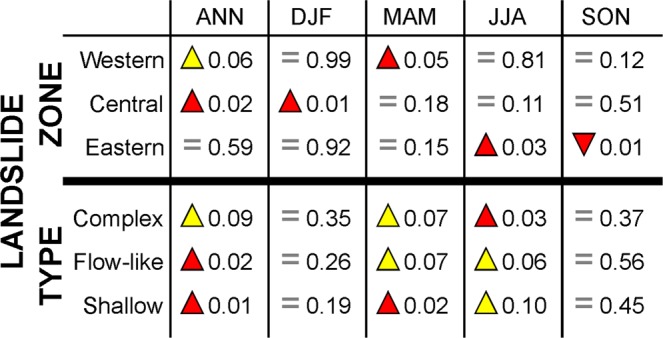
P-values of Mann-Whitney U test showing differences between years with and without landslide activity based on annual (ANN) and seasonal (DJF = winter; MAM = spring; JJA = summer; SON = autumn) precipitation totals, regions and landslide types. Red up = significantly higher precipitation during event years; yellow up = higher precipitation, but no significant difference: 0.05 < p < 0.10; equal sign = no significant difference; red down = significantly lower precipitation sums in event years.

Slight differences in triggering conditions also exist between shallow, flow-like, and complex landslides. Not only do complex landslides occur less frequently (recurrence interval: 12.9 yrs) than their flow-like and shallow counterparts (8.3 and 7.5 yrs, respectively; Fig. S5), we also observe significantly larger annual precipitation sums in years with reconstructed shallow and flow-like landslide activity (p = 0.01 and 0.02, respectively). In the case of complex landslides, differences between event and non-event years are not significant (p = 0.09). Above-average spring precipitation has the largest effect on shallow landslides (p = 0.02), whereas larger-than-normal summer precipitation seems to be the main driver of activity in complex landslide bodies (p = 0.03). Spring and summer precipitation seems to be equally important for the release of flow-like landslides (Fig. 4). In addition, landslides of the western part of the study region are most likely triggered during spring precipitation events. Interestingly, activity in its central parts probably responds to higher winter precipitation (p = 0.01), whereas no significant differences are observed during spring and summer. The eastern region shows the best correspondence with significantly higher summer precipitation sums (p = 0.03), but also with significantly lower autumn precipitation (p = 0.01; Fig. 4).

### Precipitation trends and landslide frequencies

**Figure 5:**
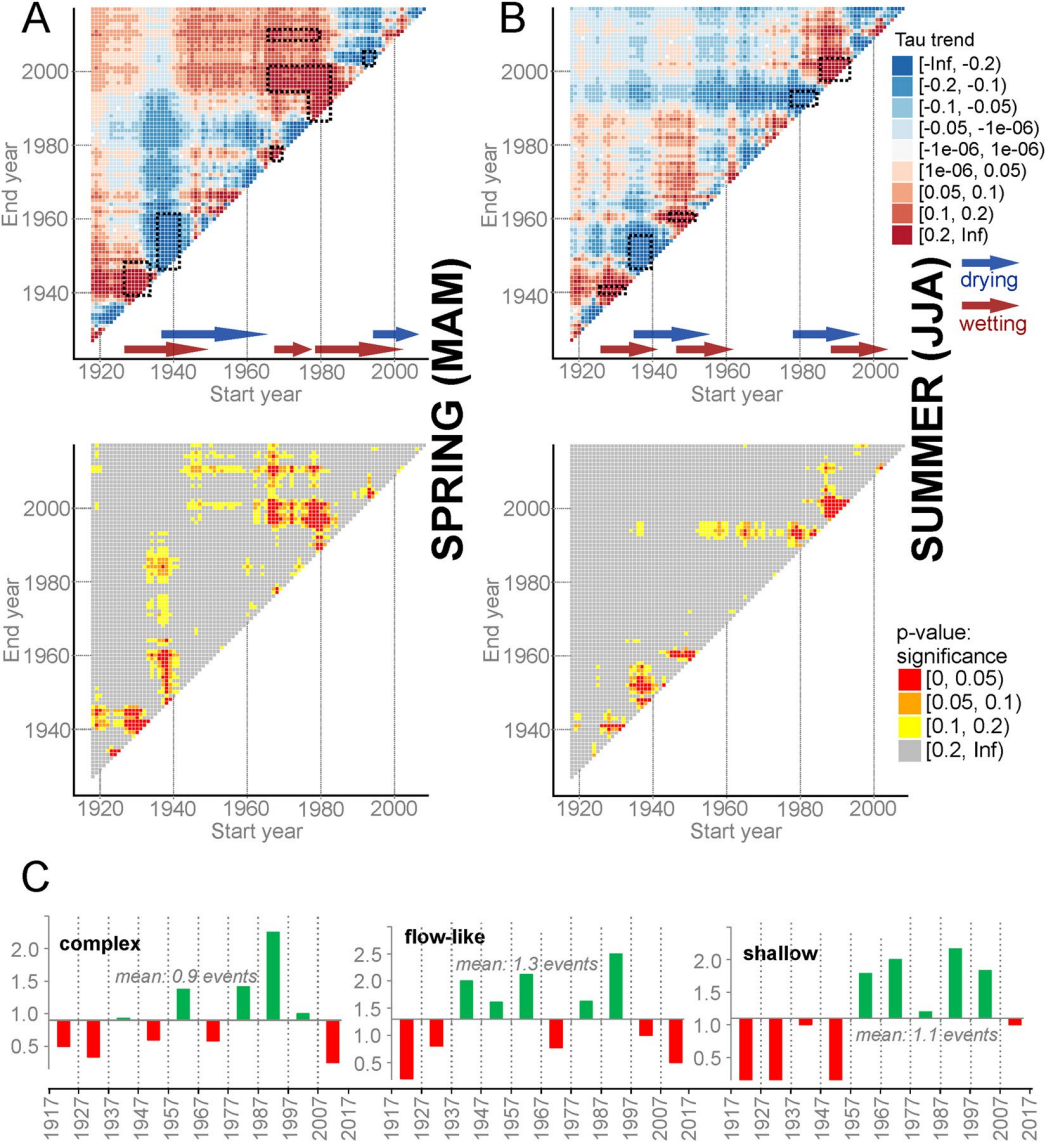
Comparison of precipitation trends with frequency of landslide activity. Decadal trends of spring (**A**) and summer precipitation (**B**) with significance levels based on the Mann-Kendal trend test (black-bordered polygons show the most significant drying or wetting periods); (**C**) decadal frequency of landslide activity based on landslide types.

Precipitation did not change significantly over the last ~100 yrs, neither in terms of annual nor seasonal sums – and only a slightly positive trend is discernible for winter (p = 0.06, Kendal’s τ = 0.13) and spring precipitation (p = 0.09, τ = 0.11). By contrast, decadal variability is quite remarkable both in terms of annual and seasonal precipitation totals (Figs 5, S6–S8). Periods of increased landslide activity correlate with periods of enhanced summer rainfall, especially if the latter occurs after drier years/summers. As such, dry periods during the 1930s and 1950s were followed by enhanced landslide activity in wet periods of the 1940s and 1960s. The same pattern cannot, however, be found during the intense landsliding of the 1990s. During the first half of the 1990s, a positive linear trend is observed in winter and spring precipitation, but also a significantly negative trend in summer precipitation. The strong increase in summer rainfall after 1995 converted the negative trend to positive, even in terms of annual precipitation sums. Despite negative decadal trends of summer precipitation observed from 1985 to 1995, landslide activity has increased, thereby pointing to the complexity of climate-landslide linkages.

### Dry spells and extreme precipitation enhance landslide activity

**Figure 6:**
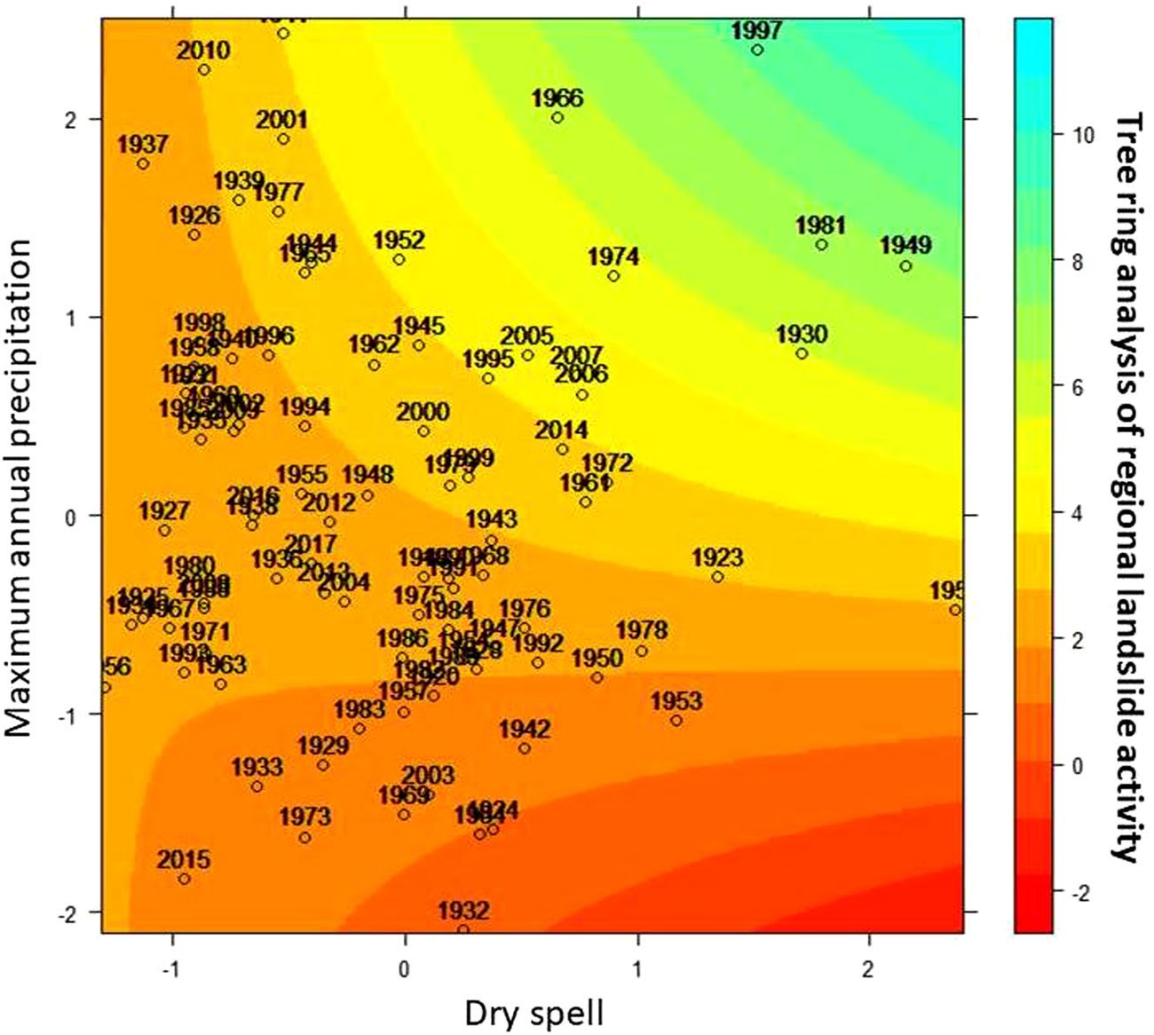
Predicted tree-ring disturbance induced by regional landslide activity is shown here as a function of maximum annual precipitation rate and dry spells by using a generalized linear mixed (GLM) model. The colour ramp indicates the predicted response of tree-growth disturbances to landslides activity. The labelled dots indicate landslide signals during specific years. The units for the maximum annual precipitation rate and dry spells have been standardized and are shown with Z-scores.

We hypothesized that increasing air temperatures have an influence on the drying rate of soils, which in turn results in the spreading of tension cracks (Fig. S2) and a weakening of soil anchorage, especially in the case where the soil is then affected by important rainfall, and ultimately favor landslide triggering. To test the hypothesis that dry spells and subsequent rainfall episodes can explain regional landslide activity over the course of the 20^th^ century, we applied a Generalized Linear Mixed (GLM) models (see methods). The GLM model suggests that the probability of landsliding increases in years with long dry spells and maximum precipitation sums (Fig. 6). The Akaike Information Criterion (AIC, see Methods) supports the statistical model accounting for the combined effect with a first-order autoregressive parameter AIC = 12175.49 against the null hypothesis (AIC = 12313.19). When compared to the individual effects of the dry spell (model parameter = −0.110; p-value = 0.328) and maximum precipitation (model parameter = 1.009; p-value < 0.000), the combined effect of both covariates is highly significant with a larger weight in model outcome (model parameter = 1.219, p-value < 0.000; number of observation = 1999; degrees of freedom = 1970; Fig. 6). Thus, our results support the positive role of maximum precipitation sums at the annual scale in triggering landslides, and that dry spells play a role in predisposing process activity by changing soil properties.

## Discussion

The spatio-temporal occurrence of landslides mainly depends on the frequency and intensity of triggers, with the latter often being site-specific and dependent on climate as well as changes thereof^6^. The significant influence of heavy precipitation on landslide reactivations has been documented repeatedly, even at regional scales^13,16,46^. Several studies also pointed to the crucial role of antecedent rainfall in triggering mass movement activity^18,47^. In the region analysed here, a stark inter-decadal variability of landslide activity can be observed over the last century, but no clear long-term trends. This is in contrast to reconstructions from the European Alps where an increasing trend of debris flows and shallow landslides has been observed over the last 100 years, mostly as a result of complex interactions between changing weather patterns triggering events, enhanced glacial retreat and permafrost degradation^7,23,48^.

Peaks in landslide activity in the study region (1940–41, 1961, 1965, 1967, and 1985) correspond with generally wet years, and most of these years were also known from historical annals^46^. Furthermore, the increased frequency of landslides recorded from 1996 to 2010 corresponds to the wet episode recorded across Central Europe. During this period, frequent floods and enhanced landsliding were reportedly caused by long-term, intense precipitation (1997, 2002, 2010)^18,46,49^.

The combined role of increased air temperatures and prolonged dry spells has been identified mostly in Alpine region where it is an important driver of rockfall occurrences^50^. By contrast, a direct link between the two parameters has not been investigated for landslide activity yet. The statistical model employed in this study supports the importance of dry spells in triggering landslide activity, at least in the study region and can be explained by the regional lithological settings. In the Outer Western Carpathians, soils and weathered bedrock contain ample amounts of smectite minerals characterised by a high sorptive and swelling capacity on the one hand and very low permeability on the other hand^41,51^. During dry spells, the amount and size of preferential flow paths (such as through tension cracks and fissures) may increase due to a shrinking of the smectite-rich soils. These new and enlarged transport conduits will then allow infiltration of water during subsequent rainfall episodes. Pore water pressure will increase rapidly in these materials and ultimately induce surface movements. Wetting/drying cycles are considered important in cracking, even in deep-seated failure movements and despite the fact that preferential flow paths will only influence the soil mantle^52^. It has also been shown that soil swelling, continuous opening/closing of the fissures, and compaction/compression forces of upslope material will result in increased pore-fluid pressure and thereby act as important drivers of activity in clay-rich, slow moving earthflows^10,53,54^. Despite the fact that forested slopes are somewhat less susceptible to occurrence of desiccation cracks than bare surfaces, the enlargement of tension cracks (Fig. S2) and fissures in specific parts of landslide bodies may increase water infiltration as well. In addition, even if trees are considered slope stabilizing agents, they may also facilitate the creation of new preferential flow paths due to the spreading of the root system (both living and decayed roots)^55^. However, mechanisms of water infiltration into a landslide body and the evolution of preferential flow paths has not been understood fully so far^52^.

According to our results, drying/wetting cycles would have favoured the initial reactivation of multiple landslides in the study area in 1939, 1940, 1972, 1985 and 1994. Landslide chronology also points to the occurrence of events during generally drier years but with concurrent extreme, yet short-lived summer precipitation (>50 mm), for instance in the years 1942, 1946, 1982, and 1988. Moreover, the extremely long dry spells in 1953–54 and 1964 were followed by extremely wet periods in 1955 and 1965, and thus point to a possible influence of longer-than-annual drying/wetting cycles (Fig. S4). In addition, results of GLM confirmed the positive anomalies of annual rainfall and dry spells in 1930, 1981, and 1997. Furthermore, during these years, long dry spells in winter/spring were followed by intense rainfall during the summer months. The 1990s were the most active period in terms of landsliding and characterised by drier summers and more humid winters/springs, first of all until 1995. Frequent alternations of dry and wet months in 1990–94 as well as the concurrent occurrence of maximum daily rainfall between 30–60 mm seem to be important factors of incipient landslide activity. In addition, the dry winter 1996/1997, in combination with slopes that have been destabilised by preceding landslide activity should thus be considered as complex preparatory factors of the enhanced landsliding in 1997 (Figs 6, S4).

In addition, we also observed an influence of above-average precipitation sums during winter and spring on landslide activity. Based on the reconstructed time series of landslides, we expect massive winter snowpack and its rapid melting in spring to be a trigger not only of shallow landslides located predominantly in the central part, but also of complex landslides located predominantly in the western part of the study region. The influence of rain-on-snow events on landslide reactivations was documented in the areas surrounding our study region^45,56^, but cannot be confirmed in this study and with the approaches used. Dry spells appear to be more important in complex landslides as differences in precipitation vary less than for the other landslide types between years with and without landsliding (Figs 4, S9). In California, such a depth-dependent response of persistently moving earthflows has been observed above all as enhanced activity of shallow landslides, but less so in deeper landslides^29^. We assume that the drying/wetting cycles in the Flysch Carpathians can be more effective in parts with abundant tension cracks and fissures. As complex landslides may represent various morphological features that are commonly observed in shallow and deep-seated landslides, the probability for the occurrence of such mechanisms could increase in the future. However, the interacting movement mechanisms of individual landslide zones can also strongly affect the timing of their reactivations^57^.

To improve linkages between landslides and climate further, it would be desirable to go beyond the construction of annually-resolved landslide chronologies by adding field monitoring to the time series of process activity^58^. In addition, radar data could improve the assessment of precipitation intensity and sums and therefore reduce uncertainties inherent to the assessment local rainfall events causing local landslide reactivations with a more regional weather station network^13^ with stations located mostly at the foothills meaning the possible underestimation of precipitation in the mountainous parts caused by orographic enhancement^34,59,60^. As the analysis presented here was performed at the regional scale and as more than half of the landslide sites are located at the foothills of the Outer Western Carpathians, errors related to the choice of stations has, however, been minimized as much as possible.

## Conclusions and Implications

The analysis of tree-ring based landslide chronologies in terms of climatic triggers and variation thereof can not only further our understanding of long-term process dynamics and the evolution of triggers, but thereby also and unambiguously help the prediction of landslide behaviour in a future greenhouse climate. In this paper, we demonstrate that mass movements are not only triggered by precipitation, but that dry spells have a significant influence on landsliding as well in the Outer Western Carpathians. This combined effect has recently been considered in persistently moving clayey landslides over the western U.S. In addition, landslide reactivations vary quite significantly between landslide types and exhibit contrasting climate-landslide linkages. In view of the anticipated increase in the frequency, intensity and duration of dry spells as well as the possibility of having more intense rainfall events, one might expect significant changes in landslide activity to occur in the study region.

## Material and Methods

Landslide activity in the study region was analysed from documentary sources (available papers, landslide database of the Czech Geological Survey, LiDAR-derived digital elevation model with a resolution of 2 × 2 m), and field survey focusing on the morphology of the landslide body with open tension cracks, visible scarps and tilted tree stems. All selected landslides (26 sites) were then divided into groups based on the type of landslide movement (flow-like, shallow, and complex landslides) and regional settings (western, central, and eastern parts of the mountain range; see Supplementary Table S1). All conifer trees (1322 tree individuals of *P. abies*, *L. decidua*, and *A. alba*) with evidence of past landslide activity (i.e. stem tilting, stem bends, exposure of root system near scarps) were sampled following standard approaches^40,61^. Two cores (one from the tilted side and one from the opposite side) were extracted from each tree with a Pressler increment borer (40 × 0.5 cm) and stored in plastic boxes. Tree position was recorded with a GPS device and supplementary information about external disturbances and social position of trees were noted. In addition, samples from reference trees (i.e. trees growing nearby the landslide sites showing no signs of geomorphic disturbances) were extracted perpendicularly to the slope at breast height.

All sampled trees were subjected to tree-ring counting and measuring with a TimeTable measuring device and PAST4 software^62^. The reference chronologies were built with Arstan^63^ using a standard double detrending procedure (with a negative exponential function, or linear regression, in the first step and a cubic spline function in the second step). Reference curves were cross-dated with disturbed samples to (i) identify pointer years, (ii) to eliminate false and missing rings in disturbed samples, and (iii) to avoid any misinterpretation related to climate-related growth signals. The onset of compression wood and abrupt growth suppression were identified as two common growth disturbances caused by landslide activity^39,64,65^. Criteria used for the consideration of growth disturbances as a result of landslide activity are described in Supplementary Table S2. Identification of event years was based on the event-response index (*I*_*t*_)^66^ with defined thresholds at 5 and 10%, and final chronologies were compiled for (i) each landslide site separately, (ii) groups of landslide types, (iii) groups based on zonal settings, and for the (iv) whole region (Fig. S5)^40^. The beginning of each chronology was determined by the year when at least 10 trees were available for analysis. The recurrence interval of landslide reactivations was then calculated as the quotient of the length of the landslide chronology and the number of reconstructed landslide events^40^.

Data series from nine meteorological stations covering the landslide region were used for the climate–landslide analysis. Stations were carefully selected based on their position, length and continuity of the data series (Fig. S3 and Table S3). Precipitation data were quality controlled based on standard methods^67^ and homogenized using the Craddock test^68^. Monthly values of the Standardized Precipitation Index (SPI) were calculated to analyse wet and dry periods^69,70^. The SPI calculation is based on long-term precipitation records which are fitted to a probability distribution before being transformed to the normal distribution. Therefore, the normal SPI is zero, negative values indicate wet months, and positive values indicate dry months. The wetting and/or drying events end as soon as the SPI becomes positive (in the case of drying events) or negative (in the case of wetting events)^70^. The combination of drying and wetting events was then compared with landslide activity. Annual and seasonal precipitation values from all stations were used to test differences between years with and without landslide activity^33,34^. Firstly, precipitation data were averaged from all stations to analyse years coming from the overall landslide chronology and chronologies based on the landslide types (under the assumption of a randomly distribution of complex, flow-like, and shallow landslides). Secondly, precipitation data were regionally averaged from stations belonging to a particular zone (western, central, and eastern) to analyse years coming from the chronologies based on zonal settings^67^. Finally, a non-parametric Mann-Whitney U test was applied to identify significant differences between precipitation during years with and without landslide activity at a significance level α = 0.05.

For trend analysis, we used the Mann-Kendall (MK) test for climate variables at different seasons, previously standardized using Z-score. The purpose of the Mann-Kendall (MK) test^71,72^ is to statistically assess if a monotonic upward or downward trend exists for the variable of interest. A monotonic upward (downward) trend means that a variable consistently increases (decreases) through time, but the trend may or may not be linear. The MK test can be used in place of a parametric linear regression analysis, which can be used to test if the slope of the estimated linear regression line is different from zero. The regression analysis requires that the residuals from the fitted regression line be normally distributed; an assumption not required by the MK test, that is, the MK test is a non-parametric (distribution-free) test.

Then we used a Generalized Linear Mixed (GLM) model to investigate linkages between tree-ring response to landslides and climate variables (i.e. dry spell and maximum precipitation) at the annual scale. In the model, tree-ring signals induced by landslide activity have been grouped by geographic zone to account for potential random effects. We also considered an autocorrelation term to account for potential long-medium-memory effects. Model selection was based on the Akaike Information Criterion corrected for small sample sizes (AICc)^73,74^. The alternative hypotheses were tested by using the delta AICc between each alternative hypothesis and the null hypothesis (i.e. AICc of the null model minus AICc of each model). To evaluate residuals in the selected model, we plotted standardized residuals against quantiles of the standard normal of the selected model and for each site analysed (see Fig. S9).All predictor variables were standardized before model fitting using a Z-score.

## Supplementary information

Supplementary material

## Data Availability

The datasets generated during and/or analysed during the current study are available from the corresponding author on reasonable request.
