# Supplementary material for: Dry Spells and Extreme Precipitation are The Main Trigger of Landslides in Central Europe
Source: Sci Rep. 2019 Oct 10;9:14560. doi: 10.1038/s41598-019-51148-2 (PMC6787093; doi:10.1038/s41598-019-51148-2)
Supplement: Supplementary file 1 — Supplementary material [file 41598_2019_51148_MOESM1_ESM.pdf]

Supplementary material

DRY SPELLS AND EXTREME PRECIPITATION ARE THE MAIN TRIGGER OF LANDSLIDES IN CENTRAL EUROPE

Radek Tichavský[1], Juan Antonio Ballesteros-Cánovas[2,3], Karel Šilhán[1,4], Radim Tolasz[5], Markus Stoffel[2,3,6]

[1]Department of Physical Geography and Geoecology, Faculty of Science, University of Ostrava, Chittussiho 10, 71000 Ostrava, Czech Republic

[2]Climatic Change Impacts and Risks in the Anthropocene (C-CIA), Institute for Environmental Sciences, University of Geneva, 66 Boulevard Carl-Vogt, 1205 Geneva, Switzerland

[3]Dendrolab.ch, Department of Earth Sciences, University of Geneva, 13 rue des Maraîchers, 1205 Geneva, Switzerland

[4]Department of Biology, Faculty of Science, University of Hradec Králové, Rokitanského 62, 500 03 Hradec Králové, Czech Republic

[5]Czech Hydrometeorological Institute, Na Šabatce 17, 143 06 Praha 4 - Komořany, Czech Republic

[6]Department F.-A. Forel for Environmental and Aquatic Sciences, University of Geneva, 66 Boulevard Carl-Vogt, 1205 Geneva, Switzerland

Contents:

Fig. S1. Morphological features of the studied landslides: (A) main scarp of a complex slope deformation with tilted tree stems; (B) deep-seated blocks of complex slope deformation; (C) distinct 2–4 m high headscarp with a deep-seated subsided block; (D) undulated surface along the tension crack with tilted tree stems. 2

Fig. S2. Distinct tension cracks and fresh openings within landslide bodies: (A) fresh hole as a part of a ca. 50m long tension crack at a complex landslide; (B) spreading of tension crack covered by soil and organic matter within a complex landslide body. 2

Fig. S3. Precipitation characteristics of the study region and completeness of meteorological records: average monthly precipitation (A), average maximum daily precipitation per month (B), and average Simple Daily Intensity Index per month (C) across the studied region; the length and continuity of the data series of each meteorological station (D). A simple Daily Intensity Index was calculated as the ratio of annual total precipitation to the number of wet days (≥ 1 mm). 3

Fig. S4. Visualisation of drying and wetting cycles based on monthly SPI, extreme daily precipitation, and reported landslide events[18,24,45,47–49] during the period 1938–2017. Possible landslide triggers in years showing I_t index and/or ratio of active landslides higher than 10% are illustrated by weather icon. The wetting and/or drying event starts when the SPI >+1 / is <-1. The wetting and/or drying event ends when the SPI becomes positive (in case of drying event) or negative (in case of wetting event). 4

Fig. S5. Individual chronologies of landslide activity based on type of landslides and landslide zonation. Black line – only one landslide site was active; red line – at least two landslide sites were active. Grey area represents the number of landslide sites covered by tree-ring chronology. 5

Fig. S6. Decadal trends of annual precipitation; significance tested with the Mann-Kendal trend test for the period 1918–2017. 6

Fig. S7. Decadal trends of winter (DJF) precipitation; significance tested with the Mann-Kendal trend test for the period 1918–2017. 7

Fig. S8. Decadal trends of autumn (SON) precipitation; significance tested with the Mann-Kendal trend test for the period 1918–2017. 8

Fig. S9. Predicted regional landslide activity as a function of maximum annual precipitation rate and dry spells for each type of landslide considered in the analysis. The figure shows similar trends regardless of the type of landslide; although with s slight difference for shallow landslides. Therefore, the latter seems to be more sensitive to maximum precipitation, independently of dry spells. This is detected by the higher values in the upper-left corner of the panel, in comparison with the complex and flow-like landslide types. 8

Fig. S10. Standardized residuals versus Quantiles of standard normal of the selected model for each site analysed. 9

Table S1. Basic morphometric characteristics of the landslide sites. 10

Table S2. Criteria for analysis of growth disturbances[37] as a response to landslide activity with defined thresholds of I_t index. 11

Table S3. Parameters of the selected meteorological stations in relation to the studied landslides. 11

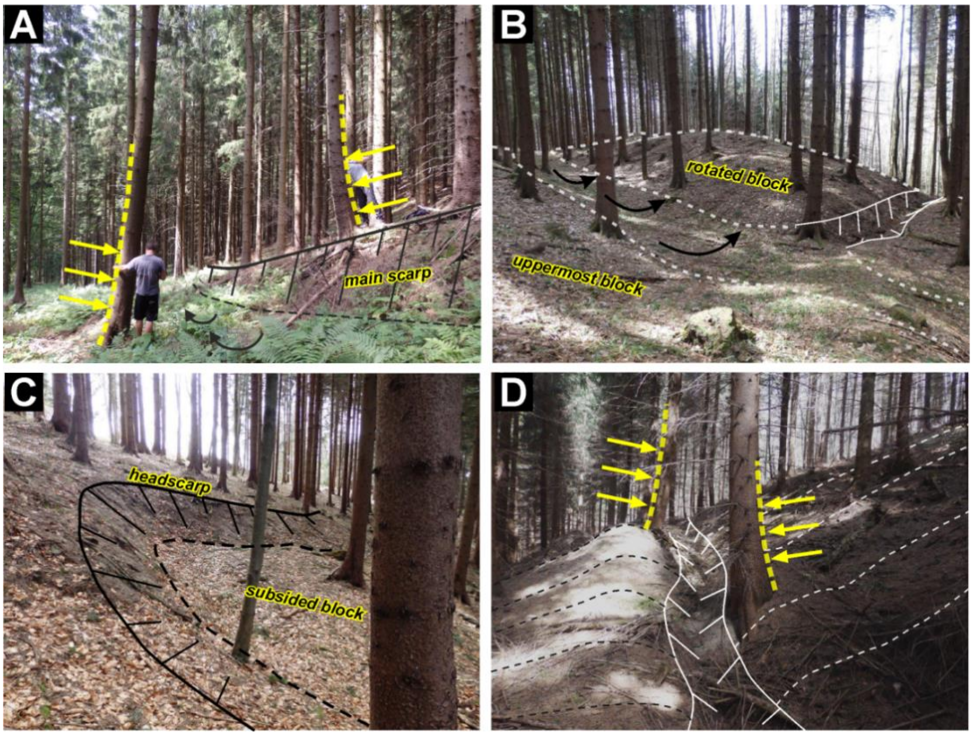

Fig. S1. Morphological features of the studied landslides: (A) main scarp of a complex slope deformation with tilted tree stems; (B) deep-seated blocks of complex slope deformation; (C) distinct 2–4 m high headscarp with a deep-seated subsided block; (D) undulated surface along the tension crack with tilted tree stems.

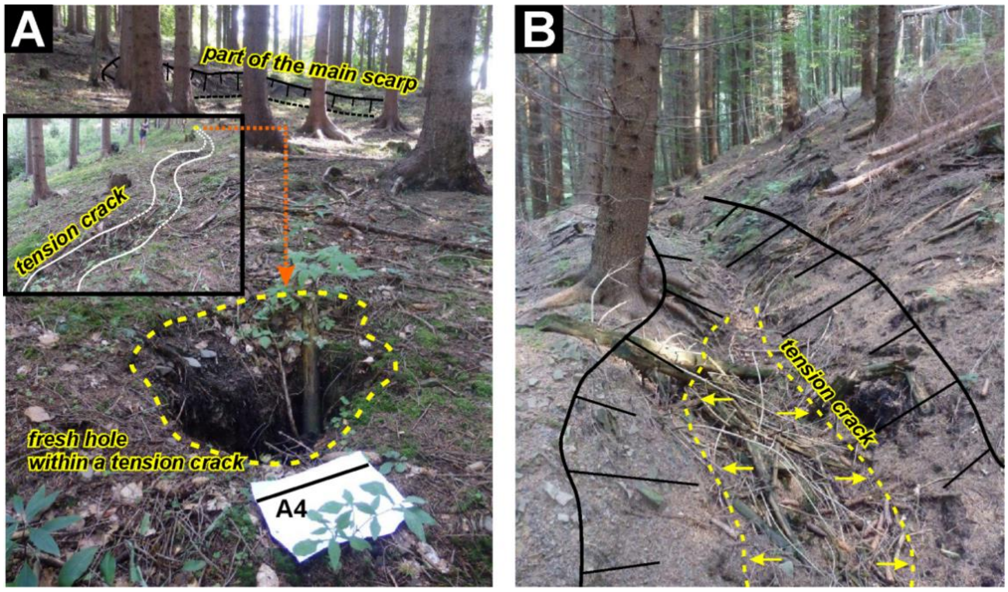

Fig. S2. Distinct tension cracks and fresh openings within landslide bodies: (A) fresh hole as a part of a ca. 50m long tension crack at a complex landslide; (B) spreading of tension crack covered by soil and organic matter within a complex landslide body.

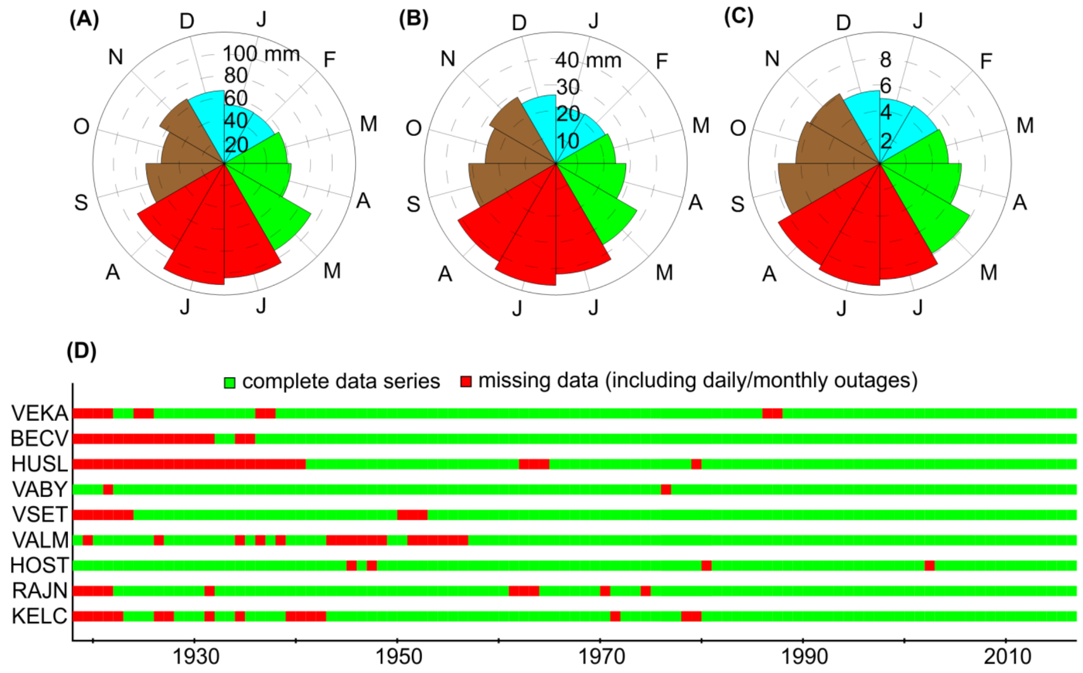

Fig. S3. Precipitation characteristics of the study region and completeness of meteorological records: average monthly precipitation (A), average maximum daily precipitation per month (B), and average Simple Daily Intensity Index per month (C) across the studied region; the length and continuity of the data series of each meteorological station (D). A simple Daily Intensity Index was calculated as the ratio of annual total precipitation to the number of wet days (≥ 1 mm).

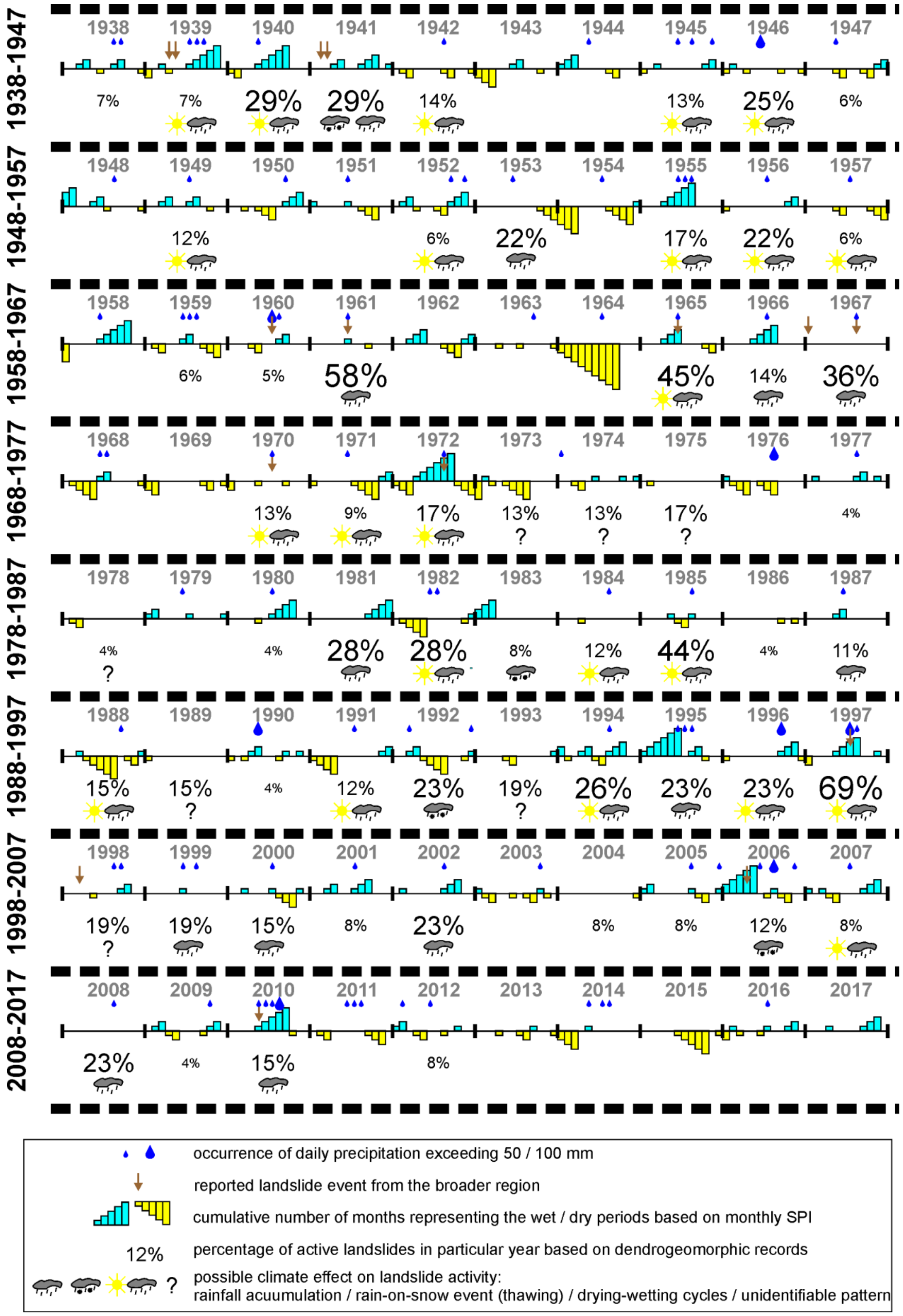

Fig. S4. Visualisation of drying and wetting cycles based on monthly SPI, extreme daily precipitation, and reported landslide events[18,24,45,47–49] during the period 1938–2017. Possible landslide triggers in years showing I_t index and/or ratio of active landslides higher than 10% are illustrated by weather icon. The wetting and/or drying event starts when the SPI >+1 / is <-1. The wetting and/or drying event ends when the SPI becomes positive (in case of drying event) or negative (in case of wetting event).

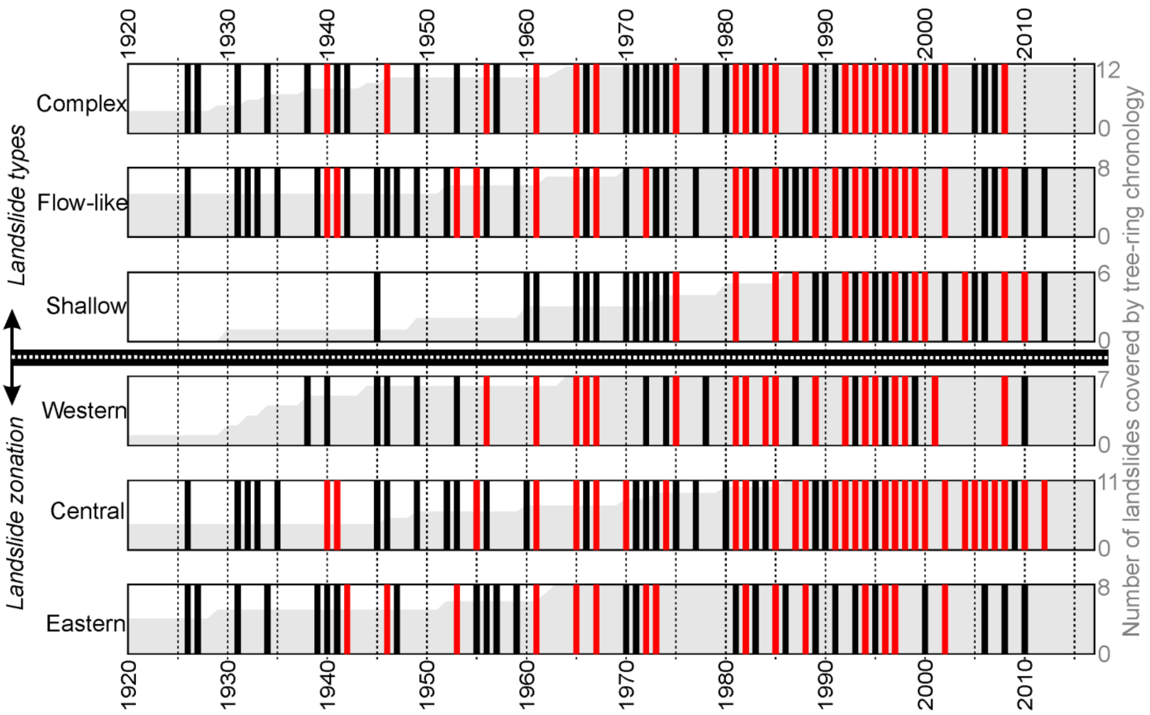

Fig. S5. Individual chronologies of landslide activity based on type of landslides and landslide zonation. Black line – only one landslide site was active; red line – at least two landslide sites were active. Grey area represents the number of landslide sites covered by tree-ring chronology.

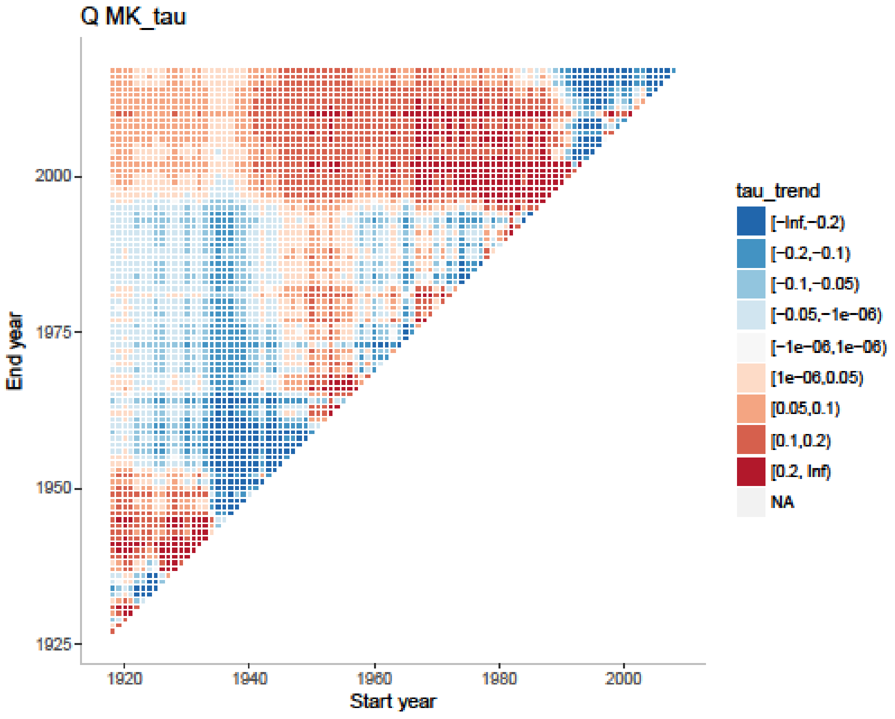

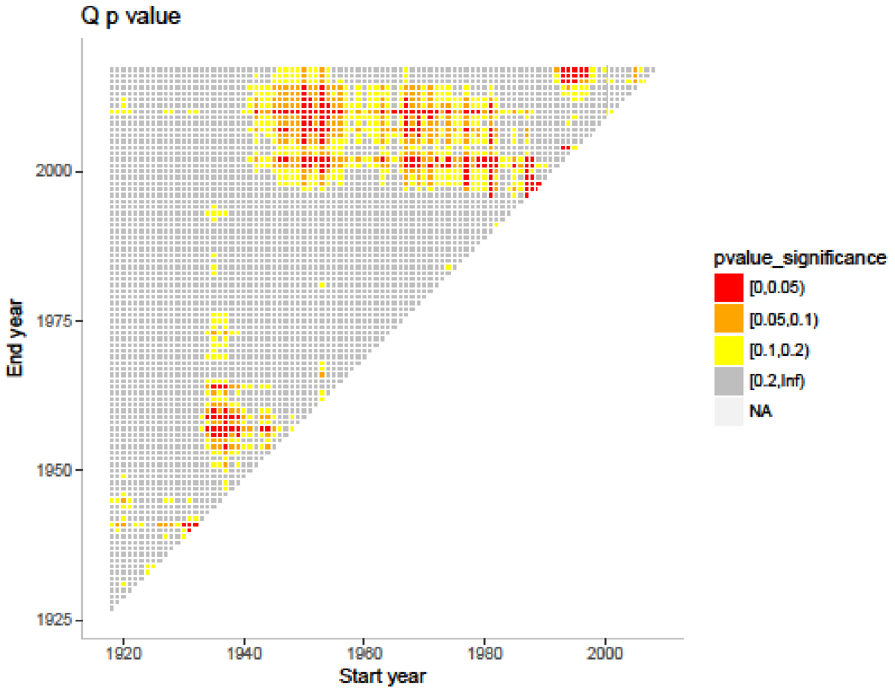

Fig. S6. Decadal trends of annual precipitation; significance tested with the Mann-Kendal trend test for the period 1918–2017.

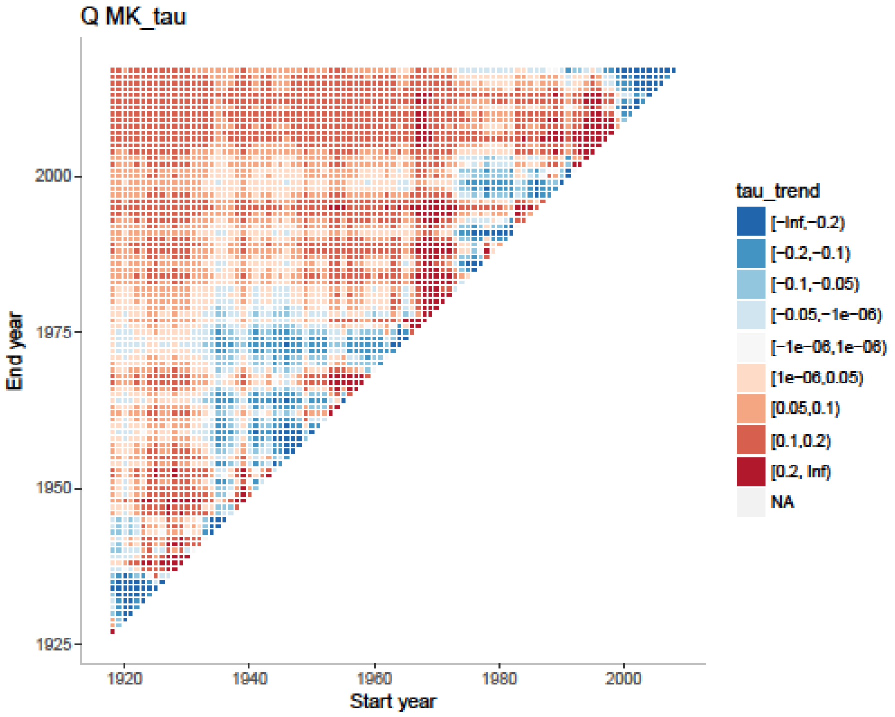

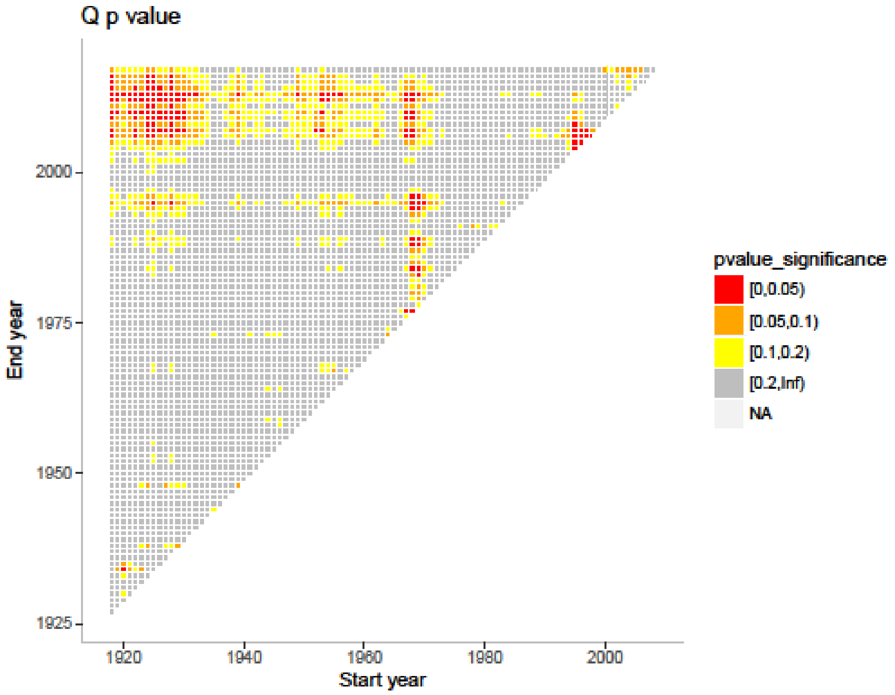

Fig. S7. Decadal trends of winter (DJF) precipitation; significance tested with the Mann-Kendal trend test for the period 1918–2017.

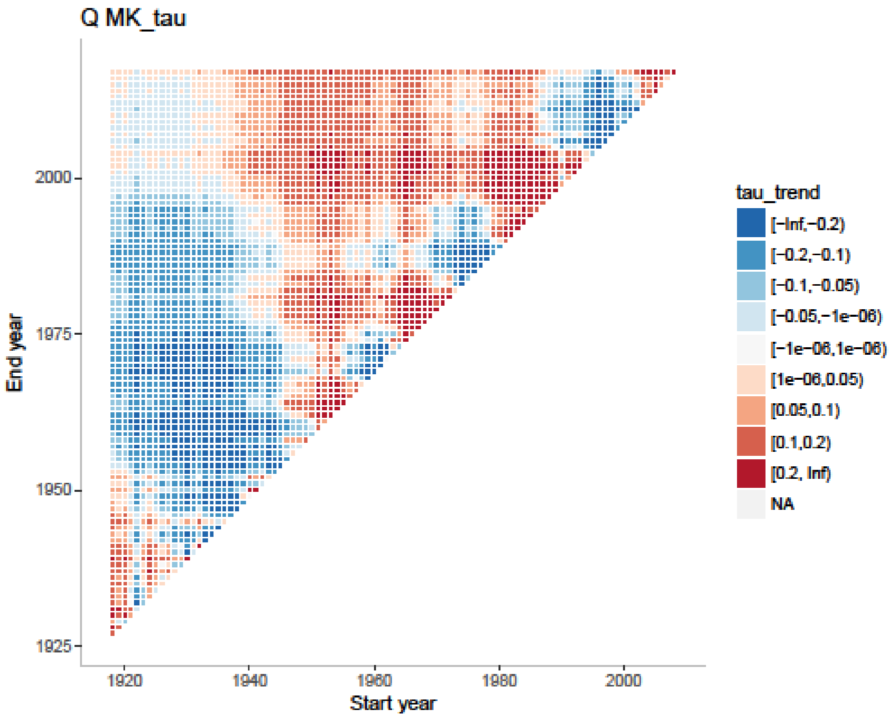

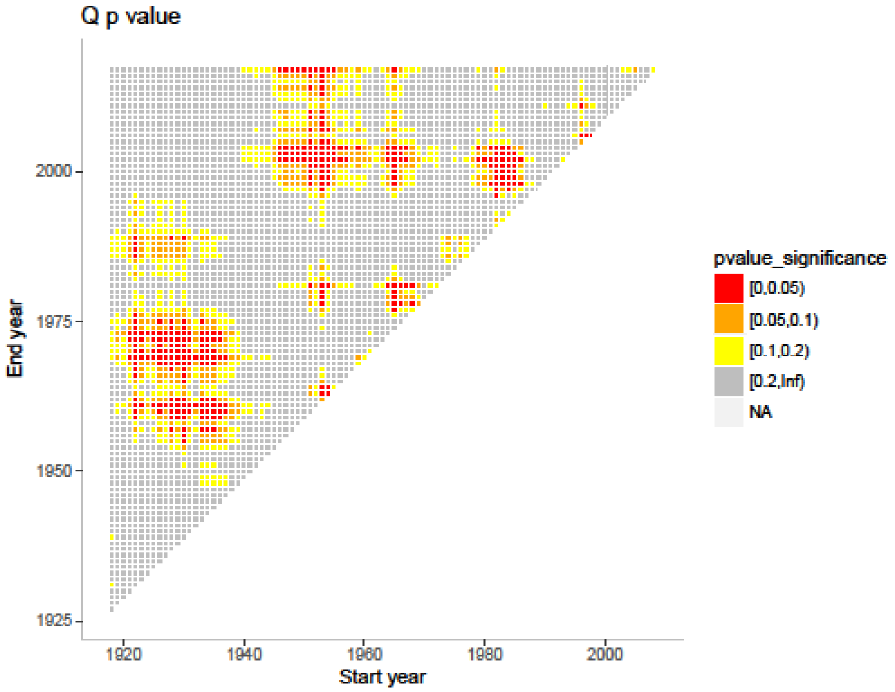

Fig. S8. Decadal trends of autumn (SON) precipitation; significance tested with the Mann-Kendal trend test for the period 1918–2017.

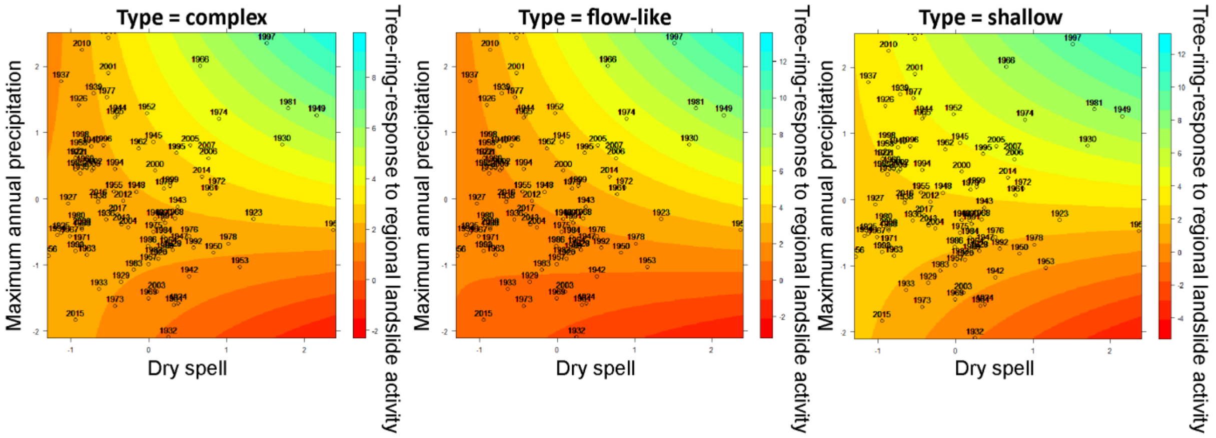

Fig. S9. Predicted regional landslide activity as a function of maximum annual precipitation rate and dry spells for each type of landslide considered in the analysis. The figure shows similar trends regardless of the type of landslide; although with s slight difference for shallow landslides. Therefore, the latter seems to be more sensitive to maximum precipitation, independently of dry spells. This is detected by the higher values in the upper-left corner of the panel, in comparison with the complex and flow-like landslide types.

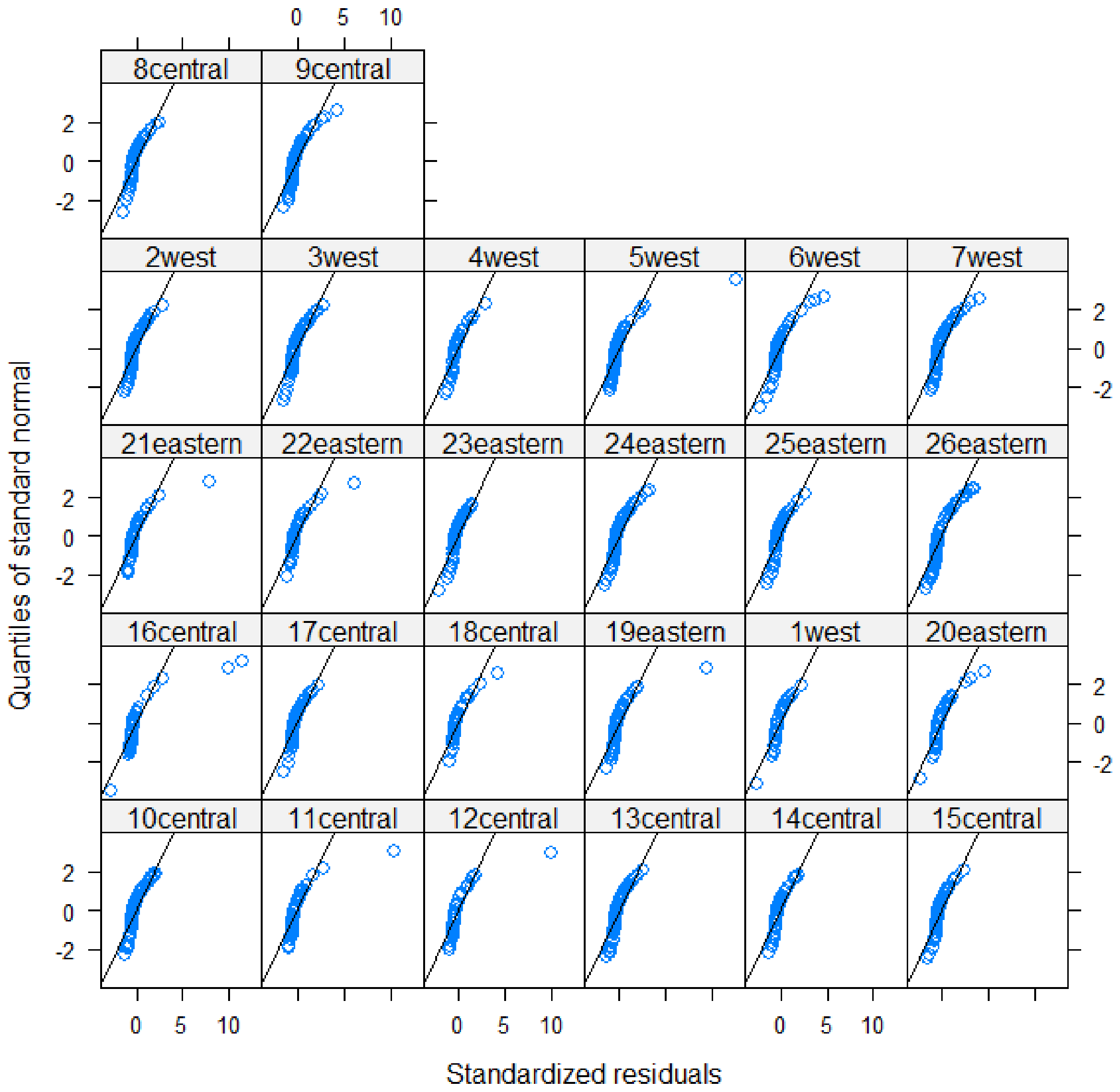

Fig. S10. Standardized residuals versus Quantiles of standard normal of the selected model for each site analysed.

Table S1. Basic morphometric characteristics of the landslide sites.

ID	Name	Type	Location (zone)	Mean altitude	Mean slope (°)	Area (m^2)
1	Loučka I	complex	western	486.9	12.9	27 810
2	Loučka II	complex	western	471.2	11.9	127 149
3	Podolí	shallow	western	443.0	13.6	54 442
4	Uvezené	complex	western	506.7	12.4	168 755
5	Kateřinice	complex	western	457.5	14.1	33 403
6	Pržno I	complex	western	435.1	13.6	93 731
7	Pržno II	complex	western	396.7	12.5	86 135
8	Růžďka	complex	central	420.0	12.0	92 597
9	Vlčice I	flow-like	central	493.8	15.4	24 430
10	Vlčice II	flow-like	central	517.8	14.6	50 107
11	Jasenka	flow-like	central	454.6	15.3	100 686
12	Dušná	shallow	central	651.6	14.2	44 682
13	Cáb III	flow-like	central	626.1	16.0	24 884
14	Cáb II	shallow	central	717.9	7.9	52 071
15	Cáb I	complex	central	723.0	10.0	39 058
16	Lubenky	shallow	central	653.1	15.7	13 200
17	Lušová I	shallow	central	603.7	22.0	10 990
18	Lušová II	shallow	central	598.4	16.8	12 070
19	Kobylská	complex	eastern	702.2	19.8	33 819
20	Soláň	complex	eastern	722.8	18.6	61 610
21	Bzové I	flow-like	eastern	621.5	15.5	15 814
22	Bzové II	flow-like	eastern	640.6	18.5	28 881
23	Jezerné	flow-like	eastern	601.7	22.4	18 512
24	Kývňačka	flow-like	eastern	706.0	18.2	11 326
25	Hluboký	complex	eastern	661.8	23.8	11 256
26	Bečva	complex	eastern	578.3	17.2	7 181

Table S2. Criteria for analysis of growth disturbances[37] as a response to landslide activity with defined thresholds of I_t index.

Growth disturbance (GD)	Parameter	Duration
Compression wood	≥50% of ring width consists of compression wood cells	≥3 years
Abrupt growth suppression	≥70% decrease in tree ring width compared to mean width of previous 4 tree rings	≥4 years

Event identificators	Calculation	Threshold
Number of growth disturbances	Total number of growth disturbances in a year *t* at the landslide site	3
I_t index	$I_t = \Sigma R_t / \Sigma N_t \times 100$ [%]<br>R_t - number of trees with a GD in year *t*; N_t - number of all sampled trees alive in year *t*	5% (probable event), 10% (certain event)

Table S3. Parameters of the selected meteorological stations in relation to the studied landslides.

ID code	Name	Altitude m (a.s.l.)	Distance to the nearest landslide (km)	Total number of landslides in 10 km buffer
RAJN	Rajnochovice	405	2.5	8
KELC	Kelč	300	5.2	4
HOST	Hošťálková	385	2.1	12
VALM	Valašské Meziříčí	334	6.3	7
VSET	Vsetín	387	1.1	13
VABY	Valašská Bystřice	465	3.5	12
HUSL	Huslenky	450	6.0	4
VEKA	Velké Karlovice	520	1.4	10
BECV	Horní Bečva	565	1.8	8
